# State-dependent foraging: lactating voles adjust their foraging behavior according to the presence of a potential nest predator and season

**DOI:** 10.1007/s00265-015-1889-x

**Published:** 2015-04-01

**Authors:** Thilo Liesenjohann, Monique Liesenjohann, Lenka Trebaticka, Janne Sundell, Marko Haapakoski, Hannu Ylönen, Jana A. Eccard

**Affiliations:** 1 Department of Animal Ecology, University of Potsdam, Maulbeerallee 1, 14469 Potsdam, Germany; 2 Department of Biological and Environmental Science, Konnevesi Research Station, University of Jyväskylä, P.O. Box 35, 40014 Jyväskylä, Finland; 3 Metapopulation Research Group, Department of Biological and Environmental Sciences, University of Helsinki, FIN-00014 Helsinki, Finland

**Keywords:** *Myodes glareolus*, Optimal foraging, *Sorex araneus*, Nest protection, Seasonality, Interference

## Abstract

Parental care often produces a trade-off between meeting nutritional demands of offspring and the duties of offspring protection, especially in altricial species. Parents have to leave their young unattended for foraging trips, during which nestlings are exposed to predators. We investigated how rodent mothers of altricial young respond to risk of nest predation in their foraging decisions. We studied foraging behavior of lactating bank voles (*Myodes glareolus*) exposed to a nest predator, the common shrew (*Sorex araneus*). We conducted the experiment in summer (high resource provisioning for both species) and autumn (less food available) in 12 replicates with fully crossed factors “shrew presence” and “season.” We monitored use of feeding stations near and far from the nest as measurement of foraging activity and strategic foraging behavior. Vole mothers adapted their strategies to shrew presence and optimized their foraging behavior according to seasonal constraints, resulting in an interaction of treatment and season. In summer, shrew presence reduced food intake from feeding stations, while it enhanced intake in autumn. Shrew presence decreased the number of visited feeding stations in autumn and concentrated mother’s foraging efforts to fewer stations. Independent of shrew presence or season, mothers foraged more in patches further away from the nest than near the nest. Results indicate that females are not investing in nest guarding but try to avoid the accumulation of olfactory cues near the nest leading a predator to the young. Additionally, our study shows how foraging strategies and nest attendance are influenced by seasonal food provision.

## Introduction

All animals are confronted with the major trade-off of gathering food to meet their energetic needs on the one hand and the costs associated with the search for food on the other hand. Potential costs include an increased risk of predation and higher metabolic needs when active (Lima 1985; Lima and Dill 1990). But, there are also benefits connected to activity, for example, increased chances to meet mating partners or to acquire and defend territories. Predictions of time investment into different activities can be made based on an equation of costs and benefits, the optimal foraging theory (OFT hereafter) and the Marginal Value Theorem (MVT), developed in the 1970s and 1980s (Charnov 1976; Kotler 1984; Brown 1988). In many studies applying the OFT and MVT, the timing of leaving an artificial food patch, or the intensity of exploiting a patch, has been used as a surrogate for perceived predation risk at the patch or while traveling between patches. These risks have been artificially manipulated by varying the cover of patches or habitats, the illumination, or the simulated presence of a predator (Brown 1988; Kotler and Blaustein 1995; Korpimäki et al. 1996; Jacob and Brown 2000; Eccard et al. 2008; Liesenjohann and Eccard 2008). On average, animals used the provisioned foraging resources less when risk was higher or when animals were further away from the shelter (Brown and Morgan 1995; Thorson et al. 1998). Additionally, animals reduced traveling to reduce the probability of meeting a predator (Norrdahl and Korpimaki 1998; Rastogi et al. 2006) and tended to shift their foraging activities to either safer habitats or safer time windows (Kotler et al. 1992; Eccard et al. 2008; Liesenjohann and Eccard 2008; Lima 2009).

In many studies on foraging behavior, however, little attention was paid to individual differences among foragers such as the sex of individuals or individual state (e.g., sexually active/inactive, breeding state of females). Nevertheless, these factors may be crucial. Mating system of a species may affect risk taking, with males from polygynous systems increasing their chances of multiple mating by risk prone activities, different from males in monogamous systems. Many small mammals have altricial offspring, permanent stationary nest sites and only the females provide parental care. This creates high nutritional demands of the mother during lactation and simultaneous pregnancy (Trebaticka et al. 2007), combined with the need to defend the offspring against nest predators or infanticidal conspecifics (Koskela et al. 2000; Ylonen et al. 1997; Rodel et al. 2008). At the same time, rodents have high predation rates on the foraging adult (Norrdahl and Korpimaki 1998). Few studies on birds and fish deal with the trade-off of foraging and nest predation (Winkelman 1996; Komdeur and Kats 1999; Sasvari and Hegyi 2000). These studies show that parents take the risk for the offspring into account when foraging or being otherwise active. Thus, strategic answers to predation risk should be evaluated according to the actual state of an animal and its offspring, reflecting on distinct stages in life history and reproductive cycle as well as the reproductive value of the offspring. Meanwhile, a trade-off between nutritional demands and offspring protection is difficult to study because often, predators are a threat to both adults and offspring. Here, we use a study system with voles and shrews, where the predator is only a threat to the offspring and the mother can successfully defend herself and her nest against the predator (Liesenjohann et al. 2011, 2013). The female strategies in temporal and spatial activities should therefore reflect adaptations to her status. Lactating bank voles (*Myodes glareolus*) were exposed to a potential nest predator, the common shrew (*Sorex araneus* (Linné 1758)), in large outdoor enclosures. Occurring sympatrically with voles across all boreal and temperate habitats, larger shrew species can have impact on the voles’ spatial behavior (Fulk 1972) and they potentially prey on vole nestlings (Ruzic 1971).

To measure the effect of the nest predator on the voles’ activity and foraging strategies, we offered a grid of foraging stations to the vole mothers around their nest sites and analyzed the distance of used foraging stations to the nest site as well and spatial distribution of foraging effort across the grid. We expected that if voles perceive shrews as a risk for their offspring, they would (i) use the stations more intensively than without shrews, because they represent a valuable and predictable food source near the nest, (ii) concentrate foraging activities on stations near the nest to stay close to the offspring, and (iii) maximize time in the nest to successfully defend the young.

The experiment was repeated in two different seasons to test if the interspecific interactions between shrews and voles change with changing seasonal availability of alternative natural food sources in the enclosure. The advancing season with falling temperatures and diminishing resources as external factor is known to alter foraging behavior and reactions to predator cues (Hayes et al. 2006; Liesenjohann et al. 2011). With decreasing food availability in autumn, animals have to extend the search area to find enough food (Ostfeld 1985), and competition for shared resources increases on an intraspecific and interspecific level. Any food resources should become more valuable due to the ending of the growing season and gradual depletion of resources and the growing energetic expenditure to gather food. In former experiments, we found that bank vole mothers decreased their summer home ranges in the presence of shrews (Liesenjohann et al. 2011) probably by staying close to the nest or to avoid encounters with shrews. On the other hand, female bank voles had larger home ranges in autumn than in summer, indicating lower food availability and, probably, an increased food competition with shrews, since reproducing bank vole females consume a high proportion of animal-based foods (Eccard and Ylönen 2006). This shows that bank vole females may run into a trade-off while the season advances: spending more and more time to gather food while leaving the nest unguarded despite the growing predation pressure and competition. Thus, we hypothesize that the foraging behavior in summer is different from the behavior in autumn: (i) Under the harsh autumn conditions, we expect the artificial food sources to become more valuable to the voles than under summer conditions and (ii) even more so in the presence of the shrews in autumn, as competition and predation pressure will be higher than in summer.

## Methods

The study was conducted in 12 outdoor enclosures of 50 m × 50 m at Konnevesi Research Station (University of Jyväskylä, Central Finland, 62° 37′ N, 26° 20′ E) in the year 2007. We ran the experiments in two seasons: in summer starting August 16 (16-h day light, temperature min 14.7 °C, max 24 °C) and in autumn starting September 13 (13-h day light, temperature min 4.8 °C, max 12.2 °C). Into every enclosure, we released three lactating females with their litter. We ran three control and three treatment enclosures per season; into the controls, we additionally released shrews. Enclosures were used only once to avoid potential delayed effects of shrew presence.

The general design of this study is additive to exclude secondary effects by intraspecific competition by conspecifics in the control enclosures. We did not add any individuals in the control enclosures as a density control (see Connell 1983 for a comprehensive discussion on additive versus replacement designs).

Ground predators were excluded by galvanized metal sheets (1 m high, 50 cm (groundwater level) dug into the ground). Predation by airborne predators was not excluded, but dense vegetation did not allow effective predation by birds of prey. Accordingly, all but one bank vole female were retrieved from the enclosures after the experiment.

### Experimental animals

Female bank voles (*M. glareolus*) were paired with a single male for 24 h in the lab. Pregnant females were kept in standard Makrolon cages with a nest box until parturition. Three females and their 1–2-day-old litters were released into one enclosure at a distance of 40 m from each other. One of the females was chosen randomly as focus animal in the foraging experiment reported here. Animals stayed in the field for 3–4 weeks, after which mothers and weaned offspring and shrews were removed from enclosures. Data of pup survival, telemetry home range, and condition of the mothers has been reported earlier (Liesenjohann et al. 2011).

### Treatment enclosures

Eighteen common shrews (*S. araneus*) per enclosure were released 2 days after the bank voles into the otherwise empty treatment enclosures in the summer round and 13 shrews per enclosure in autumn. The high initial number of shrews was chosen, because survival after relocation of shrews is unknown but likely to be low. The high number should guarantee a sufficient number of potential nest predators. The lower number of shrews used in autumn is due to the fact that it was harder to catch shrews in autumn and that in autumn (due to the colder temperatures and fewer insects), both the competition between shrews and voles and the predation pressure on nestling voles by the shrews can be assumed to be stronger.

Shrews were caught in the vicinity of the enclosures. Because shrews are very sensible to trapping stress, we checked mealworm-baited traps every hour and immediately transferred shrews into the enclosures. After the experiment, we trapped 9 ± 5 individuals (mean ± SD) per treatment enclosure of the originally released shrews and found no seasonal influence on shrew survival (Mann–Whitney test, *Z* = −0.54, *p* = 0.629). Their density after the experiment in summer was 4-fold (autumn 3.2-fold) higher than densities reported for boreal grasslands in summer (max ten animals per hectare (Hansson 1968)) The number of shrews retrapped per enclosure has been included as covariate in the analysis of variance but did not explain much of the variation. Since the foraging experiment was conducted shortly after release of shrews to the enclosures, we assume that numbers were close to the initially released numbers. We therefore used presence/absence of shrews as factor in statistical analysis.

### Design of foraging experiment

A grid of 4 × 4 feeding stations was installed around the nest box of one focus animal per enclosure, with the nest in the centre of the grid. Radiotracking immediately before the foraging trial confirmed that each female stayed close to its own nests (Liesenjohann et al. 2011); we therefore expected the focus animal alone to use the feeding stations around her nest. Distance between trays was 1 m. Each station consisted of a plastic tray (20 × 20 cm) containing 1 g of millet and 2 L of fine sand (depth in the tray about 5 cm, easily dug to the ground by bank voles (e.g., Eccard and Liesenjohann 2014)). At the beginning of exploitation, trays with this ratio guarantee an almost linear success of finding a food item in time, gradually going into a curve of diminishing returns over time, thus driving the animals into the patch-leaving decision (Brown 1988; Kotler and Blaustein 1995; Liesenjohann and Eccard 2008). All trays were covered by a lid and had two entrances of 2-cm diameter. Millet was weighed in portions of 1 g ± 0.05 g (mean ± SD) and mixed into the sand. The amount of food left in these trays after being depleted by an animal (giving-up density (GUD)) provides a reliable estimate of the perceived risk while foraging or traveling and is a well-established method to analyze foraging decisions (Brown and Kotler 2004). As these resources are not refilled, exploitation results in lower returns over time and forces the animal to decide when to leave a patch. This decision will be based on the perceived level of risk and potential energy gains at alternative sources (Brown 1988) based on the MVT (Charnov 1976).

After 3 days of pre-baiting, virtually all (91 ± 5.4 %) stations were used so all trays were sifted and mixed with 1 g of millet for the experimental run. Trays were recollected and sifted after 24 h. Although the experiment was rather short due to logistic reasons, we believe that 1 day is including several activity phases of the focus female (voles have a polyphasic ultradian activity cycle) and thus gives a representative account of the females’ spatial use of the foraging grid. All grains were dried for 3 h at 40 °C and weighed to the nearest 0.01 g. Control grains were taken from the package and dried as well; all results were corrected for the mere weight loss due to the drying process (mean loss 6 ± 1 % (mean ± SE)). Visits of trays were verified by characteristic tracks of the feet and tail of the voles. Trays attracted also insects and snails, but these did not remove grains from the trays.

### Variables and statistics

Mean GUD of the four trays near the nest was compared to the mean GUD of the 12 trays further away from the nest (paired Wilcoxon test of means within grids). The proportion of visited short-distance trays out of all visited trays (identified by digging traces and footprints in the sand) was compared to the proportion of available short-distance trays relative to all available trays (4 out of 16 = 0.25, one-sample *t* test). These two tests were performed to prove that the animals behaved according to the principles of the marginal value theorem and that the rather short distances between the trays evoke different foraging strategies at all.

The mean total intake per 24 h and the mean GUD of all used trays were analyzed with a two-way ANOVA with season and treatment as fixed factors and their interaction. For the interaction, the main effect of the shrew treatment was analyzed visually for each season separately, due to the reduced sample size. Not-normally distributed data (nr. of trays, concentration of effort, Kolmogorov–Smirnov test, *df* = 12, *p* < 0.014) without interaction was compared among seasons or shrew treatments separately, using a Mann–Whitney test. The concentration of effort is a measurement to describe investment patterns at feeding stations (Eccard and Liesenjohann 2008; Liesenjohann and Eccard 2008). It shows how much of the total intake is taken from the four most exploited trays. If all trays were exploited equally, a value of 25 % would be expected (because 16 trays were set out, four of them should make up one quarter of all used food, if exploitation was of equal distribution). One hundred percent would mean that the complete intake was taken from a single tray.

## Results

### Spatial foraging patterns

**Fig. 1:**
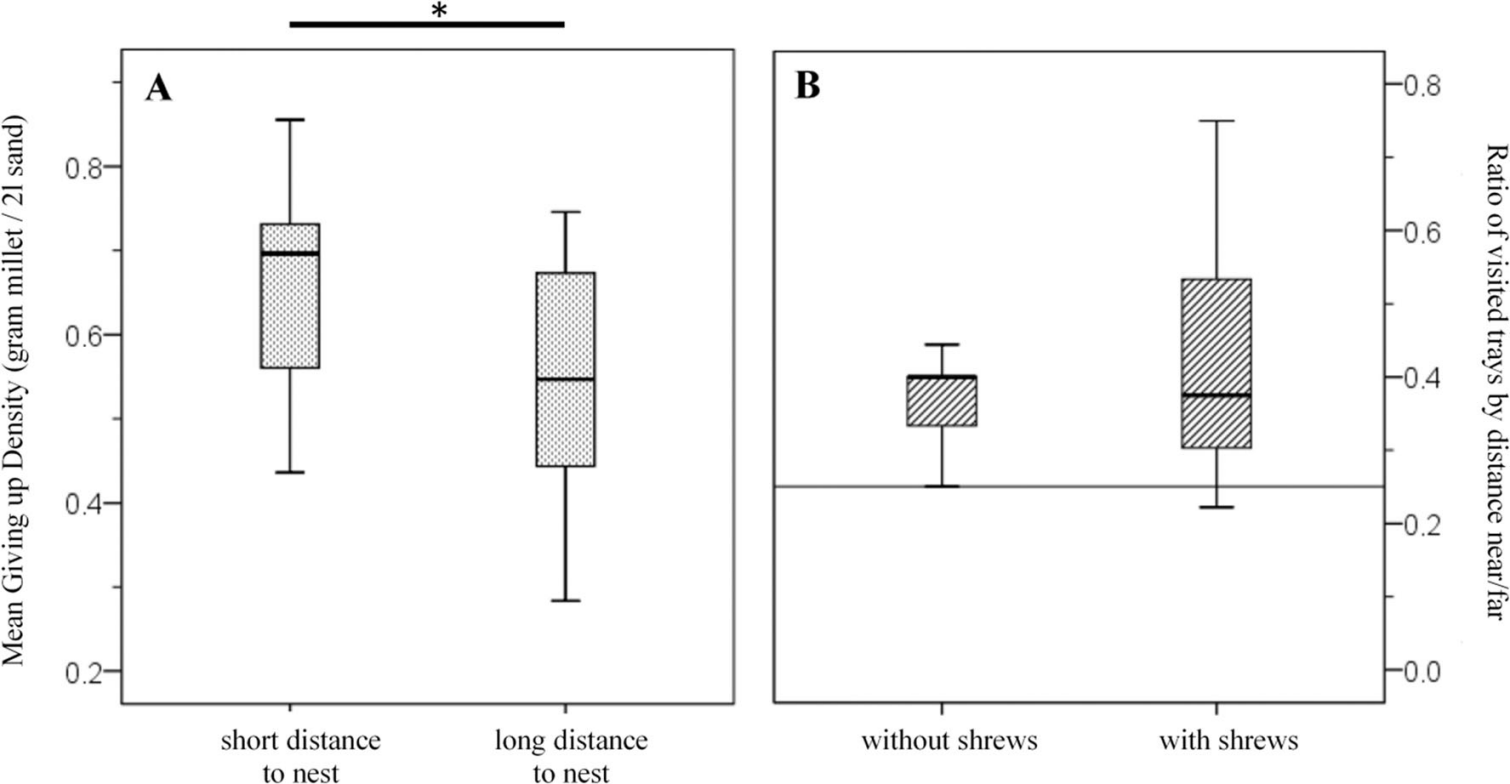
**a** Seed tray exploitation by female voles in response to tray distance to nest. **b** Effect of the shrew treatment on the ratio of near trays/far trays. In both treatments, voles used more tray near to the nest than expected by random (*reference line*). Box plots show median quartiles and range of data. *p < 0.05

Even though almost all trays were used, not all were exploited equally. Trays further away from the nest were exploited to a deeper level (lower GUDs) than those being close to the nest (mean GUD of near trays—0.65 g ± 0.13 g (mean ± SD); far trays—0.56 g ± 0.14, paired Wilcoxon test, *Z* = −2.134, *p* = 0.033; Fig. 1a). Near trays were visited more than expected by availability (0.41 ± 0.16, one sample *t* test, *T* = 3.24, *df* = 11, *p* = 0.009; Fig. 1b). The proportion of near trays visited was not affected by shrew presence (Mann–Whitney test, *Z* = 0.00, *p* = 1.00; Fig. 1b) or season (*Z* = −0.655, *p* = 0.512).

### Effects of shrew presence and season on foraging strategies

**Fig. 2:**
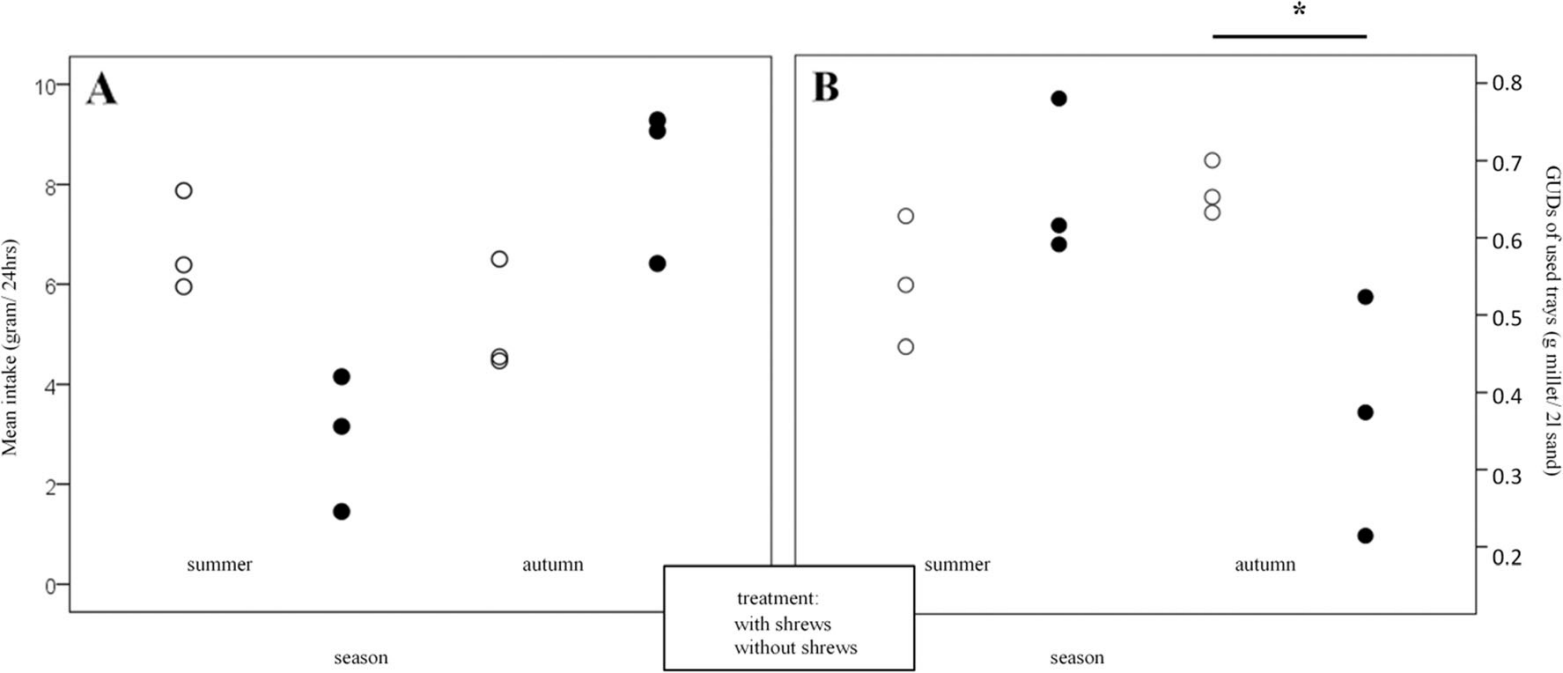
**a** Effect of the season and shrew treatment on bank vole females’ mean intake of food in 24 h. **b** Effect of season and shrew treatment on the mean giving-up densities (GUDs) of the trays exploited by the animals. Each *symbol* represents a single-day average for one female from a seed tray grid around her nest. Near and far trays are pooled.*p < 0.05

Both the total intake per grid and the mean GUDs of the used trays were influenced by a significant interaction between shrew treatment and season (Table 1). In summer, in shrew presence, all trays were depleted to lower levels than without shrews (Fig. 2a, no statistics because of low sample size) whereas in autumn, depletion levels were higher only in two out of three trays (Fig. 2a). In summer, GUDs of used trays were not affected by the treatment since GUD shrew treatments overlap with controls, while in autumn, all trays in the shew treatment were depleted to lower levels than controls (Fig. 2b).

**Table 1:** Interactive effects of season and shrew presence (treatment) on the total intake and mean GUD of used trays (two-way ANOVA) of bank vole females foraging from a seed tray grid

Dependent variable	Treatment		Season		Season × treatment	
	*F*_(1,8)_	*p*	*F*_(1,8)_	*p*	*F*_(3,8)_	*p*
Total intake per grid	1.6	0.24	1.9	0.21	17.3	0.013
Mean GUDs of the used trays	1.9	0.2	1.7	0.23	7.3	0.037

**Fig. 3:**
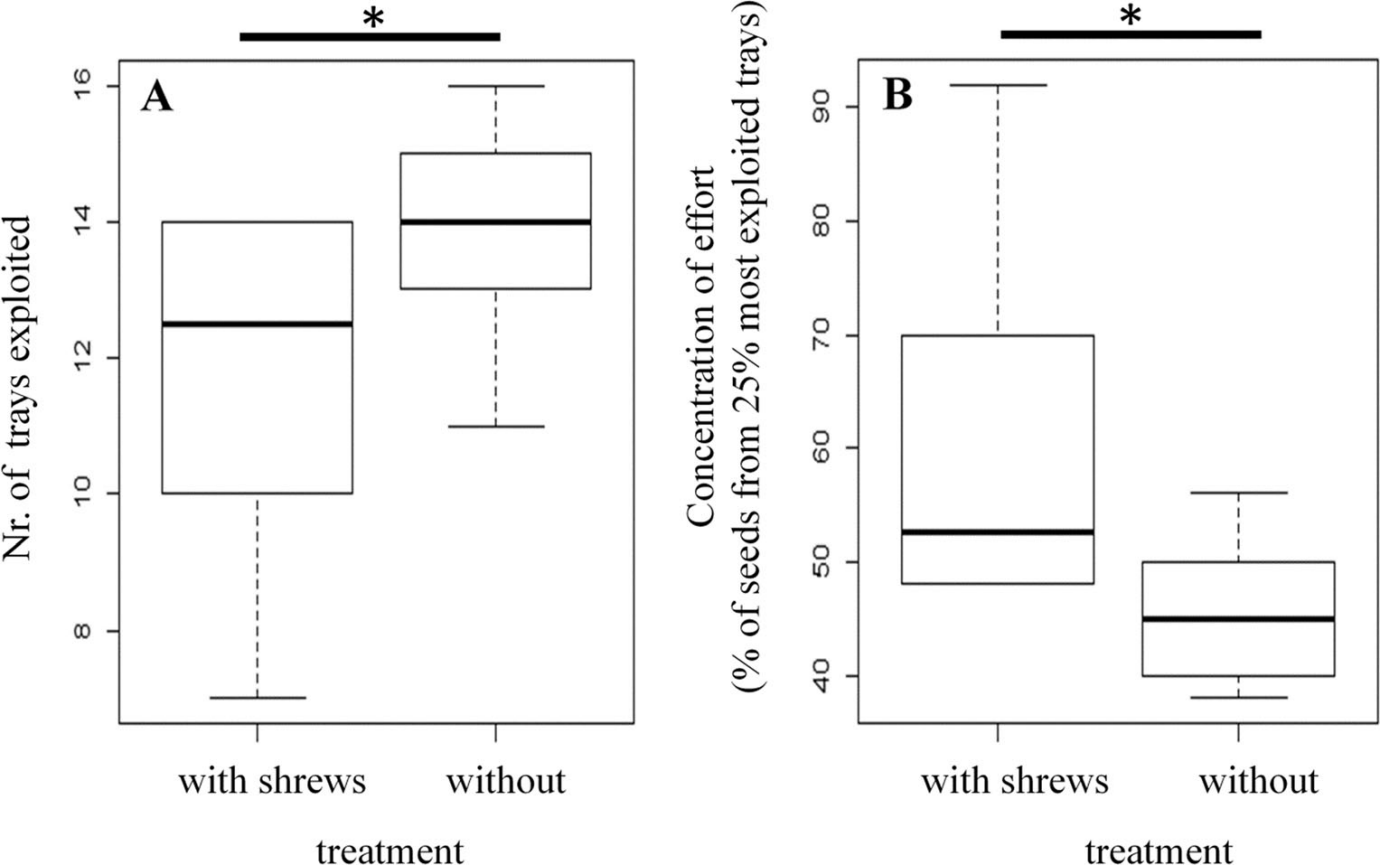
**a** Effect of shrew treatment on the number of foraging stations exploited by bank vole females. **b** Effect of shrew treatment on the concentration of foraging effort on the four most foraged trays (25 % would indicated an even distribution of foraging effort). Box plots show median quartiles and range of data. *p < 0.05

The total number of visited trays did not differ between seasons (Mann–Whitney test for the number of used trays: *Z* = −0.249, *p* = 0.803). However, with shrews present, a significantly lower number of trays were visited (mean number of visited trays without/with shrews—14.4 ± 1.1/11.2 ± 2.6, Mann–Whitney test, *Z* = −2.06, *p* = 0.039; Fig. 3a). Although the mean total intake did not differ between shrew treatments (Table 1), the animals in the shrew treatment concentrated their effort on a lower number of sources (mean % taken from the four most used trays without/with shrews—44.2 ± 7.4 %/60.8 ± 16.7 %, Mann–Whitney test, *Z* = −2.19, *p* = 0.028). Under both treatments, values were significantly different from the 25 % value expected with an even use of all trays (one-sample-t-test, without/with shrews: *T* = 5.85, *p* = 0.010/*T* = 5.62, *p* = 0.001; Fig. 3b) There was no difference in the concentration of effort between seasons (Mann–Whitney test, *Z* = −0.568, *p* = 0.570).

## Discussion

### Strategic foraging patterns under predation pressure

Our results show that lactating bank voles changed their foraging strategies according to the presence of a potential nest predator, the common shrew. Further environmental constraints were set by the changing season. Mothers reduced their intake in the presence of shrews in summer, but they enhanced the intake from the foraging stations in late autumn. Analogous to this, trays were depleted more in autumn when shrews were present, but there was no significant effect of shrew presence on the GUDs in summer. Shrew’s presence reduced the number of used seed trays, thus reduced traveling between patches, and caused the mothers to concentrate their foraging effort on a smaller number of trays. Independent of the season, mothers depleted trays far away from the nest to deeper levels than those close to the nest site. These results support our initial hypothesis that the value of a resource with a known location and known energy return is higher compared to food items outside tray that an animal has to search for. This effect is stronger in the presence of shrews, either as potential food competitors, since reproductive voles consume a large proportion of animal-based protein (Eccard and Ylönen 2006), or as well as (nest) predators and changing environmental constraints.

Small mammals in our study had to leave their nest to forage while being under the constraints of parental duties. In addition to the nutritional demands of the pups and exposure to predators while searching for food, the mothers have to deal with a potential nest predator. This provides a fitness trade-off: On the one hand, nest protection might be costly due to food deprivation (Hinch and Collins 1991); on the other hand, nest protection (e.g., against infanticidal conspecifics) can enhance the survival probabilities of the pups (Jonsson et al. 2002; Ylönen and Horne 2002). Furthermore, for the adult forager, the interaction with shrews is costly and can impair traveling and feeding activities (Rychlik and Zwolak 2006). Since voles and shrews use the same runways and tunnel systems and are of the same type of locomotion, the risk of encounter and the detection of the nest sites of the voles are high.

Behavioral strategies and life history decisions often depend on extrinsic factors like season (Kaitala et al. 1997). In our experiment, the total food intake, measured in the form of mean GUDs of the used trays, was affected by an interaction between season and treatment. In summer, the intake from the feeding stations was reduced in shrew presence (Fig. 2a), indicating the sufficient natural resources that allow the mothers to rely little on the feeding stations. This interpretation is further supported by our telemetry results with smaller home ranges in summer under shrew presence (Liesenjohann et al. 2011), indicating sufficient resources to reduce travelling. Voles reduced movements and thus decrease the probabilities of encounters with predators and interfering competitors (Norrdahl and Korpimaki 1998; Nie and Liu 2005). Yet, telemetry home ranges were still bigger than the inner grid of foraging stations, which seem to be still too close to the nest site to be accepted in the presence of shrews, as long as there is enough food in intermediate distances (near enough to secure nest protection and short foraging cycles, but far enough to keep predators unclear about the nest side). By this behavior, the amount of reliable, conspicuous, olfactory cues in the vicinity of the nest site can be reduced, especially in connection with using the far away feeding trays more intense but less frequently. High levels of urine have been proven to be a cue for predators followed by intense hunting at these places (Koivula and Korpimäki 2001). Low temperatures and lower densities of invertebrate food in autumn might force shrews to switch to alternative food sources, like vole pups, and enhance predation pressure. This was supported by our finding of reduced pup survival of the bank voles in autumn in the shrew treatment (Liesenjohann et al. 2011). But, it might as well be caused by larger home ranges and reduced food intake of bank vole mothers in shrew presence. Reduced food availability may also explain bank vole foraging patterns reported in this experiment. In autumn, intake from the trays was higher in shrew treatments, indicating interference and/or food competition between voles and shrews outside the trays (Liesenjohann et al. 2011) since reproducing bank vole females consume a high proportion of animal-based foods (Eccard and Ylönen 2006). In this case, the provided feeding stations allow keeping smaller home ranges and thus longer inactive bouts to keep the nest above threshold temperatures and prevent nest predation.

In other foraging studies providing supplemental foraging resources, these were used less when risk was high or foragers were far away from the nest site (Brown and Morgan 1995; Thorson et al. 1998). In our study, feeding stations were depleted to a lower level when the distance from the nest increased and the probability of finding an alternative source was lower in autumn *and* with shrews as a food competitor outside the trays (Fig. 1a, b). Our feeding patches provided (1) a reliable food source as compared to outside trays and (2) a place with a relatively high level of safety by providing shelter from avian predation, becoming more important the farther away the safe nest site is. These two factors become more important with increasing distance to the nest site. This foraging strategy can be explained by two different approaches to optimal decisions of lactating mothers: either parental demands do not affect optimal foraging strategies or the mother maximizes her own safety and intake depending on the distance to the nest. Alternatively, the mother tries to avoid producing intense scent marks in the direct vicinity of her nest site which lures mammalian predators as reported for lemmings (*Dicrostonyx groenlandicus*) (Boonstra et al. 1996; Banks et al. 2000; Sundell et al. 2008). It has been shown that a variety of predators uses scent marks (Cushing 1984; Hughes et al. 2010) as cues and that the ultraviolet visibility of urine attracts avian predators (Viitala et al. 1995) and thus enhances predation pressure around the nest site. Predation pressure is known to alter scent marking (Roberts et al. 2001; Hughes and Banks 2010; Sunyer et al. 2013), defecation behavior (Boonstra et al. 1996), and strategies to camouflage nest sides (Mironov 1990). In our study, this adaptation does not depend on the shrew treatment but is a general strategy promising success under all types of predation. While the mother searches for food, the probability of the pups being attacked grows over time of being alone. This should force the mother to use the stations close to the nest more intensely if she trades foraging effort for nest protection (Andersson and Waldeck 2006). Possibly, the observed foraging behavior constitutes a compromise between nest guarding and parental behavior at the nest site on the one hand (after all, the animals use a grid of feeding stations which is relatively close to the nest site) and the need to forage and avoid scent marking near the nest on the other hand, though we have not measured accumulation of scent marks where animals spend more time (e.g., Banks et al. 2000). These strategies are not necessarily an adaptation to shrews but may be a general answer to predation risk.

This latter composition of constraints is in line with the behavioral responses to shrew presence: the reduction of movement (smaller number of visited trays; Fig. 3a) and the adjustments of temporal investment at the feeding stations (concentration of effort; Fig. 3b). Fewer traveling events reduce the probability of meeting other animals (Norrdahl and Korpimaki 1998); thus, females may try to avoid costly encounters with shrews while foraging. To maintain sufficient intake from the trays, mothers invest more time and deplete the trays to a lower level when shrews are present. This makes foraging ineffective because the longer the animal depletes a non-refilling patch to gather food items, the more energy has to be invested per unit of energy returned (Charnov 1976). This indicates that higher encounter probabilities in order to maximize nest protection time are at higher costs than a loss of efficacy while foraging.

## Conclusions

Our experiment indicates that the reproductive vole females adjust their strategies to minimize the risk of leading predators to their nest sites via olfactory cues. They further accept inefficient foraging returns to avoid encounters and reduce the probability of being detected by predators. This could well explain why animals tested in natural environments often display longer patch residence times than predicted by optimality models. Foraging strategies are adapted to seasonal constraints of reduced natural food availability and higher energetic demands in autumn.

Lactating bank voles were able to adapt their foraging behavior to shrew presence. These behavioral adaptations were adjusted to season, indicating that seasonal constraints (like food availability) provide a framework within which animals express a flexible range of behavioral options. Here, we suggest that behavioral adaptations are depending not only on the individual state but also on individual demands and duties in different seasonal and risk contexts.
